# The Current Application and Future Prospects of Astragalus Polysaccharide Combined With Cancer Immunotherapy: A Review

**DOI:** 10.3389/fphar.2021.737674

**Published:** 2021-10-13

**Authors:** Fanming Kong, Tianqi Chen, Xiaojiang Li, Yingjie Jia

**Affiliations:** ^1^ Department of Oncology, First Teaching Hospital of Tianjin University of Traditional Chinese Medicine, Tianjin, China; ^2^ National Clinical Research Center for Chinese Medicine Acupuncture and Moxibustion, Tianjin, China

**Keywords:** cancer immunotherapy, traditional Chinese medicine, astragalus polysaccharide, immune checkpoint inhibitors, DC vaccine

## Abstract

So far, immunotherapy has been shown to have impressive effects on different cancers in clinical trials. All those immunotherapies are generally derived from three main therapeutic approaches: immune checkpoint inhibitors, immune cell vaccination, and adoptive cellular immunotherapy. Our research systematically reviewed a wide range of clinical trials and laboratory studies of astragalus polysaccharide (APS) and elucidated the potential feasibility of using APS in activating adoptive immunotherapy. Apart from being effective in adaptive “passive” immunotherapy such as lymphokine-activated killer treatment and dendritic cell (DC)–cytokine–induced killer treatment, APS could also regulate the anti-programmed cell death protein 1 (PD-1)/PD-L1 on the surface of the immune cells, as a part in the immune checkpoint inhibitory signaling pathway by activating the immune-suppressed microenvironment by regulating cytokines, toll-like receptor 4 (TLR4), nuclear factor kappa B (NF-κB), and mitogen-activated protein kinase (MAPK) pathways, and immune cells, such as DCs, macrophages, NK cells, and so on. In view of the multiple functions of APS in immunotherapy and tumor microenvironment, a combination of APS and immunotherapy in cancer treatment has a promising prospect.

## Introduction

Immunotherapy has been shown to have impressive effects on different cancers in clinical trials (Hodi et al., 2010; Kazandjian et al., 2016; Necchi et al., 2017). Till now, all Food and Drug Administration (FDA)–approved cancer immunotherapies included interleukin (IL)-2, interferon (IFN), dendritic cell (DC) vaccine, chimeric antigen receptor–T (CAR-T) cells, anti-programmed cell death protein 1 (PD-1)/PD-L1 mAbs, anti–cytotoxic T lymphocyte antigen-4 (anti-CTLA-4), and so on. In general, all these strategies have been divided into two categories according to their mechanisms (Sanmamed and Chen, 2018; Sanmamed and Chen, 2019).

The first category is to harm cancer cells directly, known as “passive” immunotherapy, using effector cytokines or cells in the immune system. It involves antibody-targeted treatment, adoptive immune cell therapies, and engineered T cells such as CAR-T therapy. Passive immunotherapy enhanced the immune system to much higher levels by cutting-edge means (Kantoff et al., 2010).

The second category is to activate the immune system by regulating endogenous immune functions, known as “active” immunotherapy. It involves 1) enhancement of antigen uptake, processing, and presentation to T cells by antigen-presenting cells (APCs), such as DC vaccines, with its extension of using agents such as interferons and cytokines to increase APC function; 2) the activation and expansion of naive T cells with DC vaccines, CTLA-4, or PD-1/PD-L1 inhibitor; and 3) enhancement of the effect of the immune response.

So far, the PD-1/PD-L1 inhibitor is an outstanding instance of these methods. PD-1 has been proved to be expressed in different cancers by activated T cells, B cells, natural killer (NK) cells, and bone marrow cells (Ai et al., 2020), while its ligand PD-L1 abounds in different cancers. They combine with each other on the surface of immune cells, leading to immune escape in the tumor microenvironment (TME). Therefore, there are a number of clinical trials researching PD-1/PD-L1 and other combination treatments trying to rejuvenate the regular immune function in TME.

*Astragalus membranaceus* is a type of tonic traditional Chinese medicine commonly used in Asia, and its main components such as astragalus polysaccharide (APS), astragaloside IV (AS-IV), and flavonoids could effectively improve the immune function of patients with various diseases as has been shown in previous researches (Fu et al., 2014; Auyeung et al., 2016; Zhang J. et al., 2020). It has been shown that APS mainly contained nine monosaccharides, such as Glc, Gal, Ara, Rha, Man, Xyl, Fuc, Fru, and Rib from the dried roots of *Astragalus* spp. Radix Astragali (Liu et al., 2017).

APS is often used clinically. Numerous *in vitro* and *in vivo* researches have validated that it could regulate the immune system (Fu et al., 2014; Auyeung et al., 2016; Chen et al., 2020; Zheng et al., 2020). Its immunomodulatory effect involves factors such as cytokines like ILs, tumor necrosis factors (TNFs), growth factor, and so forth, and immune cells like macrophages within TME (Zheng et al., 2020). Recently, the powerful immunomodulatory activities of APS for immunological checkpoints have attracted a lot of attention. In clinical and laboratory studies, APS has been shown to improve the effect of immunotherapy (Tsao et al., 2021). APS has been shown to drastically suppress the expression of PD-L1 genes and proteins in tumors and prevent the development of melanoma (Wang et al., 2014). This indicates that APS may regulate PD-1–PD-L1 signaling pathways, enhancing the antitumor capability of immune cells. The underlying mechanism of APS on this immune response remains a mystery. However, it has been well-known that TME plays a crucial role in the activation of PD-1–PD-L1 signaling pathways. Meanwhile, many studies have confirmed the multiple regulatory effects of APS on TME; therefore, in this review, we elucidated the effect of APS on TME and its potential ability to enhance that effect of immunotherapy.

### Astragalus Polysaccharide Combined With immune Checkpoint Inhibitors in Clinical Trials.

CTLA-4 and PD-1 inhibitors are two of the commonly used immune checkpoint inhibitors (ICIs) because they can activate and proliferate tumor-specific T cells in TME. Despite the therapeutic effectiveness against the CTLA-4 and PD-L1/PD-1 signal pathways, only a tiny number of patients have showed long-term responses. The main explanation for that is the severe immune-suppressive TME and decreased antigen presentation capacity in TME compromised the proliferation and survival of infiltrating T cells, and weakened the immune system from preventing additional T cells from migrating into tumor focus (Chen and Mellman, 2017).

The emergence of immune checkpoint blocking has revolutionized the management of advanced non–small cell lung cancer (NSCLC) (Sui et al., 2018). A high neutrophil-to-lymphocyte ratio (NLR) after the ICI treatment was associated with poorer OS. Fifty-three lung cancer patients were treated with ICI combined with APS, and the result showed that APS could normalize the NLR in patients receiving ICI combination treatments, and prolong the overall survival from 25.4 months (control group) to 26.1 months (APS group) (Tsao et al., 2021). Moreover, in another trial with 23 advanced cancer patients treated with ICI, the retrospective analysis presented real-world data of cancer-related fatigue, cachexia, or poor performance status after APS injection. The APS group showed better tolerance of ICI and remarkable Overall survival (OS) and Progression-free survival (PFS) outcomes (Yuan et al., 2021; Lai et al., 2019). In addition, it should be noted that APS can be considered as a novel nanocarrier for the delivery of hydrophobic drugs in chemotherapy and immunotherapy. APS nanomedicine boosted an overall antitumor immunity and caused the radiation-induced abscopal effect, with the ability to prolong the survival rate of tumor-bearing mice, inhibiting the growth of the primary tumor subjected to radiation as well as the secondary tumor distant from the primary lesion. The systematic antitumor immune responses and the immune memory were enhanced in mice after the treatment (Pang et al., 2019).

Based on the above analyses, APS could be used as a potential adjuvant in tumor immunity in ICI immunotherapy. Further researches of these combination administrations should be conducted in the future to further boost each drug’s benefits.

### Astragalus Polysaccharide Combined With Adoptive Immunotherapy

Over the last four decades, the promising development of adoptive immunotherapy has uncovered various therapeutic techniques in which immune cells are modified and prescribed to eliminate malignant cells. These techniques include tumor-infiltrating lymphocytes, NK cells, DCs, cytokine-induced killer (CIK) cells, DC-CIK cells, and lymphokine-activated killer (LAK) cells.

DCs, as innate immune cells, are the most potent APCs in the body, able to ingest, process, and present antigens, thereby promoting the stimulation of primary T cells. CIK cells induce or directly lead to apoptosis in cancer cells. During this process, cytokines like IL-2 and IFN-γ are released. CIK cells exhibit traits fast multiplying, high antitumoral efficacy, pan-cancer regulation, and great identification capacity. In biological immunotherapy, DCs paired with CIK cells can successfully ameliorate autoimmunity of cancer patients, hence boosting their survival rate and survival time. Thus, CIK cells or DCs cocultured with CIK cells comprising the CIK/DC-CIK cell treatment has emerged as an essential immunotherapy (Li et al., 2018). Many clinical evidence have shown that APS injection combined with CIK cell therapy can control tumor progression in patients with advanced NSCLC and breast cancer, improving the immune function of patients and the symptoms of qi-deficiency syndrome with no obvious adverse reactions. In the control group (CIK cells group), CIK cells were transfused into the vein (100 ml each time, once every W1,3,5 and totally five times, the total number of cells >1 × 10^10^/ml). In the combined group (APS injection and CIK cells group), the CIK cell treatment was combined with APS injection, intravenous drip (250 mg qd, 10 days). Ten days is one cycle; both groups received the second cycle of treatment a month later (Zhang, 2011; Zhang et al., 2018b; Table 1).

**TABLE 1:** Effect of APS on adaptive immune therapy.

Cell line	Dosage	Effect of APS on adaptive immune therapy	Ref
SKov3 cell	APS 100 mg/L, 3 d	CIK cytotoxicity↑	Xu et al. (2011)
Hela cell in female BALB/c mice	APS 250,500,1000 mg/L, twice a week, 3 w	CIK cytotoxicity↑	Huo and Shan (2016)
Eca-109 cell	APS 100 mg/L, 7 d	DC-CIK cells cytotoxicity↑	Wang et al. (2016)
pDC cell	APS 50,100, 200 mg/L, 1 d	IFN-α↑, TNF-α↑, and IL-6↑ by pDC; CD11c↑, CD80↑, and CD86↑	Liu et al. (2010)
A549 cell	APS 100 mg/L, 2 d	IFN-γ↑, IL-12↑, CD40↑, CD80↑, and HLA↑ in DCs, cytotoxicity of DC-CIK cells↑	Zhang et al. (2009)
LAK cell, Hela cells	APS 1000 mg/L, 3 d	Dead and apoptotic LAK cells↓	Yang and Zhao (1998)
K562 cells	APS 100 mg/L, 2 d	IFN-γ↑, IL-12↑, CD40↑, CD80↑, and HLA↑ in DCs, cytotoxicity of DC-CIK cells↑	Chen and Zhang (2013)
Kunming mice with S180 cell tumor	APS 100 mg/L, 2 d	CD86↑, CD80↑, in DC,TNF-α↑, and IL-12↑	Qiu et al. (2015)
DCs-CIK cell; Hela cell	APS 300 mg/L, 3 d	CD3^+^CD8^+^, CD3^+^CD56^+^, TNF-α, and IFN-γ in DCs-CIK; CD14↓, CD80↑, CD83↑, CD86↑, CDllc↑, HLA-I↑, and HLA^−^II↑ in DCs; TNF-β↑ and IFN-γ↑	Gong and Ju (2016)
K562 cells	APS 1000 mg/L, 3 d	LAK cytotoxicity ↑	Wang et al. (1995)
BALB/c mice with S180 cell tumor after CTX treatment	200 mg/kg, 8 d	NK cells↑; IL-2↑, IL-3↑, IL-4↑, IL-6↑, and IFN-γ↑	Weng et al. (2003)
HL-60 cells	15 mg/ml, 2 d	MICA expression↑ in HL-60 cells, sensitivity of HL-60 cells↑ to NK cells	Zeng et al. (2012)
Splenic DCs	APS 50,100,200 mg/L, 1 d	CD11c (high) CD45RB (low) DCs↑, Th2 to Th1↑, T lymphocyte immune function↑	Liu et al. (2010)
Spleen of BALB/c mice	0–200 mg/L, 7 d	Macrophage cytotoxicity↑, NK cell cytotoxicity↑, IgG↑ in supernatant	Wang et al. (2008)
pDC cell from CML patients	APS 50,100, 200 mg/L, 1 d	IL-6↑, TNF-α↑ secreted by pDC in treatment-naive CML patients, IFN-α↑, IL-6↑, TNF-α↑ secreted by pDC in remission stage patients	Liu (2009)

### Astragalus Polysaccharide Combined With Vaccine

The inhibition of immune response by the cancer microenvironment has been identified as a major barrier in effective tumor therapies. Apart from blocking immune checkpoints, DC-derived vaccines are acknowledged as a highly prospective method in cancer treatment. DCs are a special type of APCs that link innate and adaptive immune function, generating an appropriate immune response by uptaking, processing, and expressing antigens of tumors with the major histocompatibility complex (MHC) I and II. Then, they get into the secondary lymph tissue and activate naive cancer antigen-specific CD4^+^ and CD8^+^ T cells, differentiating these into effector cells. By activating DCs, there is a chance to induce the efficient cancer-specific immune response coordinated by cytotoxic T cells and other cancer-specific immune cells (Shortman and Liu, 2002; Darvin et al., 2018; Azarov et al., 2019; Kong et al., 2019; Wculek et al., 2019). At present, the FDA has authorized sipuleucel-T, which consists of blood cells enriched for APCs such as DCs. In addition, DC vaccines have been proven to be effective in ovarian cancer, liver cancer, lung cancer, melanoma, and glioblastoma immunotherapy *in vivo*. Clinical trials with ovarian cancer and hepatocellular cancer patients have confirmed the safety of DC vaccines (Carreno et al., 2015; Ott et al., 2017; Keskin et al., 2019; Zhang X. et al., 2020; Chen et al., 2021; Ding et al., 2021; Teng et al., 2021).

Though few clinical trials demonstrated the efficacy of APS in the DC vaccines in patients, several experiments have shown that patient's DCs can be characterized and amplified by APS *in vitro*. Some data showed that APS could be used as a helpful enhancer in the DC-based vaccination for cancer immunotherapies (Zhu et al., 2017). Liu et al. (2009) found APS-treated plasmacytoid DCs (pDC) secreted more IFN-α, IL-6, and TNF-α than the untreated group of CML patients at the remission phase, suggesting that APS can promote the function of pDC from CML. The mechanism might be related to promotion of antitumor cytokines IL-12 and TNF-a by APS (Jing et al., 2014; Qiu et al., 2015).

## Mechanism of Astragalus Polysaccharide Combined Immunotherapy

### The Potential of Astragalus Polysaccharide as an Adjuvant in Immune Checkpoint Inhibitors Therapy

#### Astragalus Polysaccharide Affect the Expression of PD-L1 in Tumor Microenvironment

Clinical studies have reported that overexpression of PD-L1 is associated with poor prognosis in multiple types of tumors (Gu et al., 2017; Zhao P. et al., 2019; Xie et al., 2019; Carlsson et al., 2020). According to several researches, as an effective method when joined with other cancer therapies, APS reduces PD-L1 expression in TME and plays an essential part in immunotherapy (Chang HL. et al., 2020).

Previous studies have shown that APS may substantially inhibit melanoma development *in vivo* and lower PD-L1 expression in TME, indicating that APS’s antitumor mechanism is also related with the PD-1–PD-L1 signaling pathway. In tumor-bearing mice, APS could also boost T lymphocytes proliferation and enhance the release of IL-2 and IFN-γ in the peripheral circulation (Wang et al., 2014). Chang et al. (2020) found that the isolated single-chain fragment variable (scFv) S12 from APS showed the finest binding ability to PD-1, inhibiting PD-L1–based T cell exhaustion. Furthermore, there were no substantial differences between the effect of ixabepilone and APS in tumor inhibition combined with the PD-1 inhibitor, suggesting that APS prevents tumorigenesis or progress with increased T cell activation, enhancing the synergistic effect with anti–PD-L1. Another study found that APS improved chemotherapy by reducing PD-L1 expression in TME via the protein kinase B (Akt)/mammalian target of rapamycin (mTOR)/ribosomal protein S6 kinase beta-1 (p70S6K) pathway. It could also reduce inflammatory-related cytokines and M2 macrophage proportion, induce DC maturation, and improve the T cell–based antitumor function, demonstrating that APS is a feasible supplement in immunotherapy (Huang et al., 2019).

Based on the facts presented above, it is expected that APS can be utilized in concert with PD-1 to promote immune cell infiltration and boost therapeutic outcomes.

#### The Effects of Astragalus Polysaccharide on Immune Cells Constituents of Tumor Microenvironment

In the TME, cancer cells have selected inhibitory ligands and their receptors that modulate T-cell effector function to enhance tumor tolerance and avoid immune system eradication. In recent years, pharmacological modulators of these pathways along with many other immune cells and cytokines have been extensively studied and used as new immunotherapeutic agents for the treatment of cancer. Through our review, we found APS has various effects on the immune cells in the TME. Tumor-associated macrophages (TAMs) are macrophages in the TME. Generally, macrophages include M1 and M2 types, which take tumor inhibitory and tumor promotion roles in TME, respectively. M1 generates reactive oxygen species (ROS) and pro-inflammatory cytokines, such as IL-2, IFN-γ, TNF-α, and IL-1β, which play essential roles in killing tumor cells. By contrast, M2 produce anti-inflammatory cytokines, such as tumor growth factor (TGF)-β and IL-10, promoting cancer progression.

As for macrophages, a study proved that APS can significantly increase the polarization ratio of M1/M2 macrophages in NSCLC cell lines, regulating inflammatory response in the TME (Bamodu et al., 2019). Wei et al. (2019) showed that this function is related to IL-6, TNF-α, iNOS, and CXCL10 through the NOTCH signaling pathway. Another study showed that APS can activate macrophages and release NO and TNF-α via the activation of toll-like receptor 4 (TLR4) and nuclear factor kappa B (NF-κB)/Rel, with the inhibition of cancer growth (Lee and Jeon, 2005). Furthermore, APS may greatly boost spleen lymphocyte proliferation and phagocytosis in mouse peritoneal macrophages, increasing the production of IL-2, TNF-α, and IFN-γ in peripheral blood (Li et al., 2020).

The role of B cells in tumor progression and antitumor immunity is only beginning to be understood, partially due to their low numbers in the TME compared to T cells. Tumor-infiltrating B cell (TIB) were found to be present in tumor-draining lymph nodes and in tumor-associated tertiary lymphoid structures. TIBs can have both tumor-promoting and antitumor functions. In another study, as for B cells, APS effectively stimulated the proliferation of splenic B cells in C3H/HeJ mice via membrane Ig in a TLR4-independent process. Meanwhile, monoclonal antibodies against TLR4 interrupted APS binding to macrophages, demonstrating a direct communication between APS and TLR4 on the macrophages surface (Shao et al., 2004).

For T cells, APS could apparently inhibit S100 sarcoma growth, increase CD4^+^ T cells expression and IL-2 production, and decrease CD8^+^ T cell and IL-4 levels in peripheral blood (Zou et al., 2012). In addition, APS dramatically prolonged survival in mice and suppressed the development of radiation-treated primary tumor. DCs are central regulators of adaptive immune responses, providing antigen presentation and ligands for co-stimulatory receptors, as well as a suitable cytokine milieu for activation, differentiation, and effector functions of T cells. In addition, APS could enhance the maturation of DCs and increase the membrane expression of MHC. Another investigation found that an APS-related immune reaction was primarily characterized by DC activation, featured with DC maturation and its improved antigen presentation ability via the TLR4 pathway. Mature DCs moved to the lymph nodes and resulted in the augmentation of the ratio of CD4^+^ T/Treg and CD8^+^ T/Treg. Similarly, another research showed APS made splenic DCs differentiate to CD11c (high) CD45RB (low) DCs and activated T lymphocytes with transfer from Th2 to Th1 (Liu et al., 2011; Shao et al., 2006).

For Treg cells, APS might inhibit the tumor growth partly by decreasing Treg with a lower TGF-β and IL-10 mRNA expression in the spleen (Sun et al., 2013). Furthermore, *in vitro*, APS inhibited the development and proliferation of CD4^+^CD25^+^Treg cells dose-dependently and time-dependently. In Hepatic cell carcinoma (HCC) TME, APS suppressed Treg cells by repairing cytokines imbalance and inhibiting FOXp3 expression. SDF-1 was critical in the Treg recruitment in the TME. APS might impede Treg cell migration by affecting SDF-1 via the CXCR4/CXCL12 pathway (Li et al., 2012). For NK cells, a study showed that APS could promote the transformation of mouse peritoneal macrophages to M1 type under the stimulation of tumor cells and increase the production of TNF-α and NO. APS could also improve the antitumor effect of NK cells. The IFN-γ or granzyme B–positive cells in total NK cells were higher than those in the control group (Chai et al., 2019). Myeloid-derived suppressor cells (MDSCs) take part in tumor development in a variety of ways, including angiogenesis stimulation, epithelial-to-mesenchymal transition (EMT), and pre-metastatic niche establishment. They act as a negative regulator of T-cell and NK-cell activities in the TME by hijacking immunological checkpoint pathways. Treatment with APS inhibited tumor development in MDSCs by reducing the proportion of Gr-1^+^CD11b^+^MDSCs and limiting vascular endothelial growth factor (VEGF) and IL-10 expression (Chai et al., 2012).

#### The Effects of Astragalus Polysaccharide on Immune Cell Constituents of Tumor Microenvironment

The TME is an inflammatory environment that contains a variety of inflammatory and regulatory mediators, such as cytokines IL-1β and IL-6, as well as chemokines and ROS. In the TME, cytokines are extremely important. TGF-β and IL-10 are the most well-known immunosuppressive mediators in the TME. DCs and T cells are both suppressed by IL-10. Meanwhile, IFN-γ, TNF-α, IL-2, and IL-1β play crucial parts in killing tumor cells according to previous researches (West et al., 2013; Garris et al., 2018; Balta et al., 2021). Furthermore, another study showed that intratumoral DC’s IL-12 cytokine signaling leads to anti-tumor immunity, and the antitumor cytokines IFN-γ and IL-12 are stimulated by immunotherapy. T-cell–DC interaction involving IFN-γ and IL-12 is required for effective anti–PD-1 cancer treatment. HIF-1α expression is largely regulated via various cytokines, which involves the cascading of several growth factors and oncogenic cascades (Noman et al., 2014).

APS-activated macrophages, which increase the concentration of nitric oxide (NO) and TNF-α, acting as an upstream inhibitor of tumor cell proliferation with G1 cycle blockage and modulating genes associated with apoptosis, prevent cancer cells growth (Li et al., 2019). In H22-bearing mice, APS could effectively inhibit solid tumor growth and increase body weight and phagocytotic function of macrophages. Furthermore, APS treatment could increase the release of IL-2, IL-12, and TNF-α and decrease the IL-10 level in the serum (Yang et al., 2013; Zhao et al., 2019).

In tumor-bearing mice, APS has synergistic tumor suppressive effects with Adriamycin (ADM). This may be related to its ability to enhance the level of IL-1α, IL-6, IL-2, and TNF-α, reduce IL-10, and reduce MDR1 mRNA and p-gp expression levels (Xiao et al., 2009; Tian et al., 2012; Lai et al., 2017). In patients with lung cancer, the expression of IFN-γ and T-bet was strong after the treatment of APS, while the expression intensity of IL-4 and GATA3 decreased significantly (Wei and Tian, 2003; Liao et al., 2020).

In addition, APS could effectively inhibit the growth and metastasis of Lewis lung cancer in mice and LOVO cells (Tie, 2019), enhancing the activity of normal human lymphatic endothelial cells and inhibiting the protein expression of VEGF and EGFR in tumor tissues (Lee et al., 2020).

APS could revert the increased invasion capacity of the HUVECs co-cultured with SCC7901 cells and reduce the high levels of matrix metalloproteinase (MMP)-2, MMP-9, LOX, SNAIL protein, and vimentin mRNA expression and decreased E-cadherin mRNA expression. The macrophage migration inhibitory factor (MIF) is an inflammatory cytokine that promotes the EMT (Youwei and Yeting, 2020). It is related to MMP-13 and AMP-activated protein kinase (AMPK). A study showed that APS reduced MMP-13 and activated AMPK in A549 and CL1-2 cells, reducing the AXL, vimentin, MMP-13, and MIF expressions. These studies revealed that APS plays an essential role in cancer therapy by suppressing and aggressively removing MIF from cancer cells.

According to these studies, we found APS could significantly suppress the levels of TGF-β and IL-10 *in vivo* and *in vitro* while increasing the levels of IFN-γ, TNF-α, IL-2, and IL-1β, regulating the growth factor VEGF, and reverting the immune-suppressed TME, hopefully to be an adjuvant drug in immunotherapy (Table 2).

**TABLE 2:** Effect of APS on ICI signaling pathway and TME.

Cell line and animal model	Dosage	Effect of APS on ICI signaling pathway and TME	Reference
Non–small cell lung cancer (NSCLC) H441 and H1299 cells	APS(3 mg/kg/d), 16 w	M1 macrophage↑, M2 macrophage↓, DCs maturation↑, T cell-mediated anticancer↑, M1/M2↑, DCs↑, CTL↑, IL-6↓, IL-10↓, NF-κB↓, CD11b↑, and CD31↓	Bamodu et al. (2019)
4T1 and CT26 cells; BALB/c mice	50 μg of APS intraperitoneal injection, 7 days	Cell surface PD-L1↓ via Akt/mTOR/p70S6K pathway	Chang et al. (2020b)
Inbred strain BALB/c mice (approximately 6–8 weeks old, female); the murine mammary carcinoma 4T1 cells and RAW264.7 cells	APS 30, 100, and 300 μg/ml, 24 h	M1↑, notch ligand↑, iNOs↑, TNF-α↑, and CXCL10↑	Wei et al. (2019)
BALB/c mice; RAW264.7 and 4T1 cells	APS 50, 100, 200, 500, and 1,000 μg/ml, 24 h	Spleen lymphocytes↑ peritoneal macrophages phagocytosis, IL-2, TNF-α, and IFN-γ in peripheral blood	Li et al. (2020a)
RAW264.7 and 4T1 cells; B6C3F1 mice	APS (10, 50, and 100 μg/ml), 24 h	NO↑, iNOS↑ through NF-κB/Rel ↑	Lee and Jeon, (2005)
RAW 264.7 cells; MCF-7 and RAW264.7 murine	APS 1000 μg/ml, 24 h	NO↑, TNF-α↑, IL-6↑	Li et al. (2019)
BALB/c mice with 4T1 allograft tumors	50 μg of APS, 7 days	M1/M2↑, Ki-67↓, CD25↑, and anti-PD-1 antibody titers, IL-2↑, IFN- γ↑ (scFv) S12 from APS	Chang et al. (2020a)
C57BL/6j (H-2b) mice	APS 10, 50, 100 and 250 μg/ml for 24 h	BM-derived DC maturation↑, CD11c↑, I-A/I-E↑, IL-12↑ in DC, DC endocytic↓	Shao et al. (2006)
H22-bearing mice	APS 100, 400 mg/kg/d, 10 d	Spleen and thymus indexes↑, macrophages phagocytotic↑, IL-2↑, IL-12↑, TNF-α↑, and IL-10↓	Yang et al. (2013)
Female Balb/c mice (6–8 weeks) with irradiated tumor. *In vitro*:NIH3T3, HUVEC, 4T1, and bone marrow–derived dendritic cells (BMDC)	APS nanoparticles (ANPs) (equal to 20 mg/kg APS). After each dose of radiation, DNP and ANP (20 mg/kg, 3 times) were injected into the irradiated tumor; *in vitro*, ANP (equal to 50 μg/ml APS) for 24 h	DC activation↑, maturation↑, antigen presentation↑ through TLR4 pathway. CD4^+^ T/Treg↑, CD8^+^ T/Treg↑, IFN-γ↑, effector memory T cell↑	Pang et al. (2019)
Fresh tissue samples from 31 patients with HCC	APS 10, 50, 100, 150, and 200 ug/ml for 24, 48, and 72 h	CD4^+^CD25^+^Treg↓ through cytokine balance and FOXp3mRNA↓in TME. Treg cell migration↓ by SDF-1↓ or CXCR4/CXCL12 pathway↓. Th2 cytokines IL-10↓ and IL-4↓, Th1 cytokines IFN-γ↑	Li et al. (2012)
C57BL/6 mice (7–8 weeks) with B16-F10 melanoma	APS 0.1 g/kg, 10 d	T cell↑, IL-2↑, IFN-γ↑ in peripheral blood, PD-1 mRNA↓ in spleen, PD-L1, PD-L2↓ in tumor	Wang et al. (2014)
S100 sarcoma mice	APS 50, 100, 200 mg/kg, 12 d	CD4 ^+^T↑, CD8^+^T↓, IL-2↑, IL-4↓	Zou et al. (2012)
Peripheral blood mononuclear cell (PBMC) of 18 cancer patients and 6 control patients	10% Astragalus injection, 48 h	IFN-γ↑, T-bet↑, IL-4↓, GATA3↓, IL-6↓	Wei and Tian, (2003)
SCID mice injected with A549 cells, lung adenocarcinoma A549 and CL1-2 cells	APS 10,40, and 160 mg/kg injected intraperitoneally	AXL↓, vimentin↓, macrophage migration inhibitory factor (MIF)↓, E-cadherin↑, MMP-13↓, AMPK↑	Liao et al. (2020)
RAW264.7 cells	APS 25, 50, and 100 μg/ml	NO↑, TNF-α↑, IL-6↑, iNOS↑, p-p65↑, p-p38↑, Jun N-terminal kinase↑ via NF-κB p65/MAPK signaling↑ pathway	Feng et al. (2021)
23 patients with metastatic disease	500 mg, 250 mg, 3 times/week, 4 w	IL-1β↓, IL-4↓, IL-6↓, IL-13↓, IL-17↓, MCP-1↓, GM-CSF↓, VEGF↓, TGF-β1↓, IFN-γ ↓, and IL-10↓ and IL-12↓	Huang et al. (2019)
RAW264.7 cells on 4T1 cells	APS 1 μg/ml, 100 ng/ml, 24 h	NO↑ and gene expressions of TNF-α↑, IL-6↑, iNOS↑ through TLR4, TLR4-related MAPKs↑, pERK↑, pJNK, phosphorylated p38↑, translocation of NF-κB↑, of IκB-α↓	Wei et al. (2016)
YAC-1 C57BL/6(H-2Kb) peritoneal macrophage	APS 10 mg/ml, 7 days	Abdominal macrophages to M1, TNF-α↑, IL-12↑ NO in macrophage↑; NKG2-mediated NK cell function↑	Chai et al. (2019)
C57BL/6J with Lewis lung cancer cell	100, 50, 25 mg/kg, qd, 3 w	VEGF↓ and EGFR↓ in tumor tissues	Zhao et al. (2019a)
BALB/c, nude, and NOD SCID mice HCT116	50 μg of APS, 7 d	Neutralize VEGF in mice; scFv 4E effectively inhibited human umbilical vein endothelial cells induced by VEGF *in vitro*	Lee et al. (2020)
SCC-25 BALB/c nude mice	APS 10, 25, 50 mg/kg, tid, 1 w	Ki67↓, VEGF↓, pJAK2/pSTAT3↓ by JAK2/STAT3/c-myc	Deng et al. (2019)
LOVO cell	APS 1.5 mg/ml, 48 h	VEGF-C mRNA↓, HLEC↑	Tie (2019)
C57BL/6 male mice with B16-F10 melanoma	APS 150, 300 mg/kg, 0.2 ml gavage, qd, 14 d	IL-10↓, TGF-β↓, VEGF↓, IFN-γ↑, TNF-α↑, Gr-1^+^ CD11b^+^myeloid derived suppressor cells	Chai et al. (2012)
EPC in 20 patients	APS 10 mg/kg, 7 d	IL-12↓, IL-6↓ VEGF↓, TREM-1↓, EGFR↓ in p-AKT-AKT-VEGF signal pathway	Sun et al. (2020)
SGC7901 cells; HUVEC cells	APS 10 mg/ml, 48 h	Invasiveness↓, MMP-2↓, MMP-9↓, LOX↓, Snail↓ protein and vimentin mRNA expression↓, and increased E-cadherin mRNA expression↑	Youwei and Yeting (2020)
BMSCs	APS 50 μg/ml, 42 d	α-SMA↓, FAP↓, STAT3↓, p65↓ through pathways of IL-6/STAT3 and TNF-α/NF-kB	Zhang et al. (2018c)
BMSCs and A549 cells	APS 100 μg/ml, 16 h	IL-1β↑, TNF-α↑, IL-6 ↑, NO↑. TIPE2↓, pERK↑, pJNK↑, p-P38↑ with the activation of the MAKP signaling pathway	Zhu et al. (2018)
MSC; A549	APS 50 μg/ml, 7 d	Expression of acetylated H4K5↓, acetylated H4K8↓, and acetylated H3K9↓, RAS↓, ERK↑, NF-κB p65↑, p-p56↓, TP53↑, caspase-3↑ in MAPK/NF-κB pathway	Zhang et al. (2019)
Hepatocellular carcinoma H22-bearing mice	APS 100, 200, 400 mg/kg, 15 days, qd	Spleen and thymus indexes↑, IL-2↑, IL-6↑, and TNF-α↑, Bax↑, Bcl-2↓	Lai et al. (2017)
H22 cells; S100 cells	APS 50, 100 mg/kg, 10 days, qd	IL-2↑, IL-6↑, IL-12↑, TNF-α↑	Xiao et al. (2009)
Caco2 cell	APS 400 μg/ml, 24 h	APS could suppress the LPS-induced MyD88-TRAF6 activation by a TRIF-dependent way, IL-1 β↓, IL-8↓, and TNF-α↓	Liqing (2017)
H22 tumor-bearing mice	APS 100 mg/kg, 24 h	IL-1α↑, IL-2↑, IL-6↑, TNF-α↑, IL-10↓, MDR1 mRNA↓, P-GP↓	Li et al. (2020b)
Female BALB/c mice and female C3H/HeJ mice	APS 50, 100, 200 mg/kg, 0.2 ml, qd, 10 days	APS activates B cells via membrane Ig in a TLR4-independent manner, peritoneal macrophages activation IL-1β↑ and TNF-α↑in BALB/c mice	Tian et al. (2012)

#### Astragalus Polysaccharide Affects the Pathway of Tumor Microenvironment in Immune Checkpoint Signaling

Feng et al. (2021) studied the immunoregulatory effects of APS in RAW264.7 cells via the activation of the NF-κB p65/MAPK signaling pathway. It enhanced the protein levels of phosphorylated p65, p38, Jun N-terminal kinase, and extracellular signal-regulated kinase. Then, APS enhanced cytotoxicity potential of RAW264.7 cells on 4T1 cells with more NO and cytokines and upregulated gene expressions of TNF-α, IL-6, and iNOS. Furthermore, studies showed that TLR-4 may take part in the process in which APS promotes the cytokine production of RAW264.7 cells. Another study supported the ability of APS to activate TLR-4–related MAPKs, demonstrating that APS activates macrophages through the TLR-4–MAPK–NF-kb pathway (Wei et al., 2016; Zhu et al., 2018).

MyD88 and TRIF are well-known downstream signaling pathways of the TLR4 signaling pathway, and their divergence leads to the immune function diversity of the TLR4 pathway. APS stimulated the key nodes in TLR4-MyD88–dependent signaling pathway, including MyD88, TRAF-6, TLR4, AP-1, and NF-κB in the spleen cells to regulate their immune function (Liqing, 2017; Li Y. et al., 2020).

In a clinical trial, the general situation of patients after APS treatment was significantly improved. APS could significantly reduce the levels of IL-6, IL-12, VEGF, and EGFR to protect cells from esophageal carcinoma (EPC) injury through the pAKT–AKT–VEGF signal pathway in a study by Sun et al. (2020). Another study showed APS inhibits the growth of SCC-25 xenograft tumors, increases the survival rate, and downregulates the expression levels of Ki-67 and VEGF by inhibiting the JAK2/STAT3/c-myc signaling pathway (Deng et al., 2019). As for the Bone mesenchymal stem cells (BMSC), APS had inhibited the conversion of BMSCs to TAFs induced by IL-6 and TNF-α, related to the regulation of the pathways of IL-6/STAT3 and TNF-α/NF-kB. APS improved cell morphology, decreased cell proliferation, and cell cycle disruption. The MAPK/NF-kB pathway, TP53, caspase-3, acetylated H4K5, acetylated H4K8, and acetylated H3K9 were included in this regulatory process (Liqing, 2017; Zhang et al., 2018c; Zhang et al., 2019).

**FIGURE 1:**
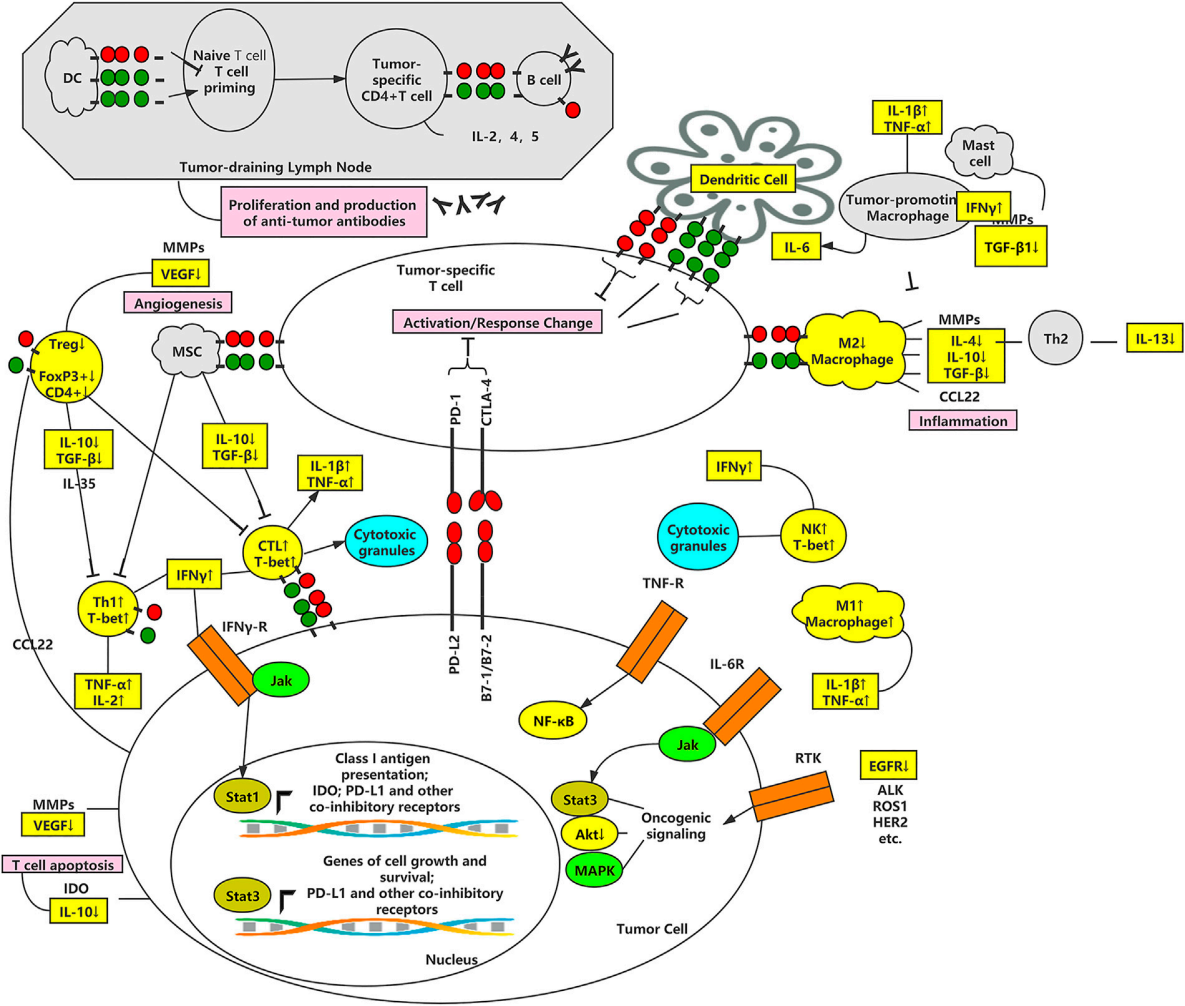
The effect of APS on ICIs signaling pathway in TME.

These discoveries are significant for us to understand the molecular mechanism of APS on TME. On the basis of the above facts, it is fair to believe APS might successfully increase the immunological activity of the TME along with the ICIs (Figure 1).

### Astragalus Polysaccharide Increases the Efficacy of Immunoadoptive Therapy

Many studies have suggested that APS could increase the expression of CD40, CD80, and HLA-DR on the surface of DCs (Peng and Luhang, 2006). It could increase the DCs and DC-CIK cells proliferation. APS-treated DCs have the most typical structures and phenotypic markers of DCs (CD86 and HLA-DR). Liu et al. found that APS increased the amount of IFN-α, TNF-α, and IL-6 generated by pDC and promoted the differentiation of pDC (Chen et al., 2009). These results suggested that APS can enhance human humoral and cellular immunity through the pDC pathway and can be expected to be an effective adjuvant in pDC-based immunotherapy with the effect of TLR9 on pDCs. Moreover, after being processed by APS, DC-CIK cells were more powerful than ordinary DC-CIK cells in antitumor reaction. The combination of APS and DC-CIK cells led to an enhanced killing ability of A549, K562, and esophageal Eca-109 cancer cells. In addition, the cytotoxicity toward Hela and SKOV3 cells in the CIK cells combined with APS group was higher than those in the CIK cells group. *In vivo* results showed that CIK combined with APS inhibited the growth of Hela-implanted tumors significantly, and the inhibitory rate was 80.6% (Peng and Luhang, 2006; Chen et al., 2009; Liu et al., 2010; Gong and Ju, 2016).

According to these studies, APS exerts cytotoxic effects on cancer cells by synergizing the cytotoxicity of CIK cells against tumor cells. These properties of APS are beneficial to the treatment of leukemia, lung cancer, ovarian cancer, cervical cancer, and many other malignant tumors (Zhang et al., 2009; Xu et al., 2011; Chen and Zhang, 2013; Huo and Shan, 2016; Wang et al., 2016).

### Astragalus Polysaccharide Could Enhance the Cytotoxicity of Other Immunotherapy

Wang et al. (2008) showed that APS could significantly enhance activation of peritoneal macrophages and splenic NK cells. Another study revealed that APS could reduce the volume densities of the dead and apoptotic LAK cells during an immune attack toward Hela cells and enhance the antitumor activity of IL-2/LAK cells (Wang and Lou, 1992; Wang et al., 1994; Wang et al., 1995; Yang and Zhao, 1998). APS also increased the cytotoxic ability of NK cells in S180 tumor-bearing mice. In addition, APS augmented the expression of MICA in HL-60 cells, increasing cytotoxicity of NK cells to HL-60 cells (Weng et al., 2003; Zeng et al., 2012).

As a potent adjuvant in vaccine immunization, APS could enhance the impact of the presentation of antigen by improving the performance of MHCII, facilitate lymphocyte proliferation, enhance the number of serum antibodies, and increase cytokine segregation. These effects are involved with improvement of immunity by increased IgM and IgG (Zhang et al., 2018).

It could be concluded that APS has various kinds of effects on the immune system through LAK cells, NK cells, and so on.

## Future Prospects

It is now well recognized that with the expansion of the use of ICIs, caregivers face new challenges in managing the adverse effects, such as diarrhea, fatigue, and itching, gastrointestinal complications, pancreatitis, hepatotoxicity, endocrinopathy, and so on (Wojtukiewicz et al., 2021).

On the other hand, ASP may improve the hypothalamic–pituitary–adrenal (HPA) axis and has been shown to exert hepatoprotective, hematopoietic neuroprotective, cardiovascular-protective, antidiabetic, and antioxidant effects (Si et al., 2015; Xie et al., 2016). APS pretreated mice showed a significant decrease in alanine aminotransferase, NF-kB expression, aspartate aminotransferase, and lactate dehydrogenase levels and increased antioxidant enzymes. On top of these, APS is a suitable candidate for adjuvant medicines without serious safety concerns, as they are often eaten in the diet for a long time (Liu et al., 2014; Xie et al., 2016).

As for the side effect of APS, in a randomized, double-blind study, APS injection could effectively reduce cancer-related fatigue in advanced cancer patients (310 subjects), and the efficacy of high-dose (500 mg/day) and low-dose (250 mg/day) groups of APS injection was statistically similar. Patients with higher KPS may benefit more from treatment. Five symptoms have been classified as treatment-related adverse events with over 2% incidence, such as rash, pyrexia, feeling cold, chills, and hypersensitivity. More than 90% of the reported adverse events were not related to APS treatment. However, more clinical data are needed to verify the safety of APS in cancer patients and its effect on cancer patients (Wang et al., 2019).

In light of aforementioned studies, it is expected that APS could exert more function in immunotherapy in the future. Nonetheless, there are still some limitations. Firstly, in terms of the effect on adverse events in immunotherapy, such as CTLA-4 or PD-1, the mechanism of APS remains a mystery. Thus, further studies are needed to confirm the feasibility of APS in immunotherapy-related adverse events. Secondly, it is still need to be explore about APS pharmacokinetics and its difference through gut and blood. Thirdly, the investigation about its side effects is still incomplete, which should be elaborated in more clinical trials in the future. Researches collected in this review were mostly conducted in China which might cause some limitations and bias. Therefore, more global researches should be carried out to verify the efficiency of APS. There is still a lot to be desired related to the all-round functions of APS and its indications and contraindications in immunotherapy.

## Conclusion

Our research systematically reviewed a wide range of clinical trials and laboratory studies, elucidating the potential plausibility of using APS in activating adoptive immunotherapy, as an immunological adjuvant in the future. We found that APS could play a positive part in the immune checkpoint inhibitory signaling pathways by activating the immune-suppressed microenvironment with regulated cytokines, TLR4, NF-κB, and MAPK pathways and immune cells such as macrophages, NK cells, DCs, and so on. Also, this review contributes to an understanding of APS as an adjunctive therapy to ICIs by optimizing the immunity balance in the TME and primarily elucidates the underlying mechanism of APS on the microenvironment and immunotherapy systematically.

To conclude, the combination of APS and immunotherapy in cancer treatment has a bright prospect; however, direct effectiveness and the mechanism of APS integrated with ICI on the tumor microenvironment need more confirmations and clinical researches.
